# Exposure to whole-body vibration and hospitalization due to lumbar disc herniation

**DOI:** 10.1007/s00420-018-1316-5

**Published:** 2018-05-31

**Authors:** Jens Wahlström, Lage Burström, Peter W. Johnson, Tohr Nilsson, Bengt Järvholm

**Affiliations:** 1 Department of Public Health and Clinical Medicine, Occupational and Environmental Medicine, Umeå University, 901 87 Umeå, Sweden; 2 Department of Environmental and Occupational Health Sciences, School of Public Health, University of Washington, Seattle, WA USA

**Keywords:** Low back pain, Lumbar radiculopathy, Lumbar disc disease, Sciatica, Whole-body vibration, Occupational drivers, Epidemiology

## Abstract

**Objective:**

The aim was to examine if exposure to whole-body vibration (WBV) increases the risk for hospitalization due to lumbar disc herniation.

**Methods:**

The study basis is a cohort of 288,926 Swedish construction workers who participated in a national occupational health surveillance programme from 1971 until 1992. Job title, smoking habits, body weight, height and age were registered at the examinations. Assessment of WBV were made for each of the constituent occupations by constructing a job-exposure matrix (JEM). Exposure to WBV was graded on a scale from 0 to 5. In addition, the occurrence of hospitalization due to lumbar disc herniation from January 1st 1987 until December 31st 2010 was collected from a linkage with the Swedish Hospital Discharge Register. Poisson regressions were used to estimate relative risk with 95 percent confidence intervals (95% CI), adjusting for age, height, weight and smoking, using white-collar workers and foremen as a reference group.

**Results:**

There was an increased risk for hospitalization due to lumbar disc herniation for workers in the construction industry exposed to medium to high WBV compared to white-collar workers and foremen 1.35 (1.12–1.63). When restricting the analyses to include workers 30–49 years of age at the time of the hospital admission the risk was 1.69 (95% CI 1.29–2.21).

**Conclusion:**

This study further supports that occupational exposure to whole-body vibration increases the risk for hospitalization due to lumbar disc herniation.

**Electronic supplementary material:**

The online version of this article (10.1007/s00420-018-1316-5) contains supplementary material, which is available to authorized users.

## Introduction

Low back pain (LBP) is common and believed to affect almost all of us at some point in life (Deyo and Mirza 2016). One specific entity of LBP is lumbar disc herniation. A lumbar disc herniation may cause severe pain and neurological symptoms such as numbness, tingling and/or muscle weakness in the area (muscles) innervated by the affected nerve. The radiating pain and the neurological symptoms are usually caused by compression or irritation of one of the lumbosacral nerve roots (Deyo and Mirza 2016). If the sciatic nerve is affected, the condition is also commonly called sciatica. The majority of cases slowly improve over a period of a few months (Deyo and Mirza 2016). If the symptoms are severe or the condition does not improve with nonsurgical treatment, surgery is one possible treatment option. Far from all herniated discs lead to radiating pain or neurological symptoms, studies using magnetic resonance imaging have observed that up to 70% of asymptomatic individuals have herniated or prolapsed discs that compress the nerve roots (Boos et al. 1995).

Lumbar disc herniation is believed to be caused by both individual and environmental factors. Associations between lumbar disc herniation and genetic factors (Battie et al. 2007, 2009), height (Heliövaara 1987a, b; Hrubec and Nashold 1975; Sorensen et al. 2011; Wahlström 2012), weight (Shiri et al. 2014) and smoking (Wahlström 2012; Jhawar et al. 2006; Leino-Arjas et al. 2008) have been demonstrated. Age has a strong influence on the incidence of lumbar disc herniation. Unlike many other diseases it is most common in mid-life (Wahlström 2012; Heliövaara 1989; Strömqvist et al. 2010). Occupational factors such as heavy lifting, working in bent and stooped positions have been associated with lumbar disc herniation (Seidler et al. 2003, 2009).

Operating a vehicle implies exposure to whole-body vibration (WBV) of differing intensities which are often dependent on the type of vehicle (Burström et al. 2015). Several reviews have observed associations between exposure for WBV and LBP (Burström et al. 2015; Bovenzi and Hulshof 1999; Waters et al. 2008) and in a recent review also between WBV and sciatica (Burström et al. 2015). However, to our knowledge, no large prospective studies on the association between WBV and hospitalization for lumbar disc herniation have been published. If there is an increased risk also for lumbar disc herniation, more effort should be spent in prevention measures to decrease the exposure.

The aim was to examine if exposure to whole-body vibration increases the risk for hospitalization due to lumbar disc herniation.

## Methods

According to a national collective agreement between employers and unions, construction workers in Sweden from 1968 to early 1993 were offered occupational health service through a nation wide program called Bygghälsan. Workers were invited to participate in health examinations every 2–5 years and the participation rate has been estimated to at least 80% (Bergdahl et al. 2004). Results from the health examinations were collected in a computerized register from 1971 including information on e.g., job title, smoking habits, body weight, height and age. The personal ID number for each individual, a unique number assigned each Swedish inhabitant, was used to find out whether the person was alive, dead or had emigrated by linkage with official Swedish registers. A small percentage of workers (0.15%) were not found in any of the registers, and they were excluded. In total the register contained 389,132 persons.

The Regional Ethical Review Board in Umeå provided ethical approval for the study (Dnr 2011-367-32M).

### Exposure

A construction sector job-exposure matrix (JEM) has been developed for WBV. The exposure assessment was based on a survey conducted in the mid-1970s (Anonymous 1977). Each construction occupation (trade) was studied during site visits to approximately 5 work sites in different geographical regions of Sweden, but for some exposures up to 15 work sites were examined to obtain enough information on the relevant exposures. WBV exposure was graded on a 0–5 scale by occupational hygienists and physicians and was assessed as the mean daily exposure. Level 0 is no occupational WBV, level 1 corresponded to a WBV exposure that at that time was considered ‘very low’, level 5 was considered ‘extremely high’, and level 3 corresponded to a WBV exposure that was considered ‘acceptable’. The metrics were estimated for the mean daily average WBV exposure by an occupational hygienist (LB) based on the job at the first health examination. Two job classifications were used, one including 212 jobs during the period 1971–1985/86 and one including 90 jobs during 1985/86–1993 e.g., there were different types of carpenters depending on tasks during the earlier period. For the later period, the grading of the WBV exposures was carried out by one occupational hygienist and one ergonomist on basis of the older matrix and the estimated WBV exposure changes that occurred in the cohort under the time period.

The proportion of construction workers exposed to occupational WBV was rather low. There were only 70 men with exposure level 1 and they were merged with the construction workers without occupational exposure (level 0). Workers with exposure levels 2–5 were then merged into the second group (there were 6722 men in group 2, 6398 men in group 3, 2212 men in group 4 and none in group 5).Thus, in the analyses, three exposure groups were used: foremen and white-collar workers (reference group), no or very low exposure (WBV 0–1) and medium to high exposure (WBV 2–5).

## Outcome

The occurrence of hospitalization due to lumbar disc herniation was collected from a linkage with the Swedish Hospital Discharge Register, which holds information on persons who have been hospitalized for at least one day. This register has nationwide information from 1 January 1987 and includes codes for diagnoses. We included data until 31 December 2010. To be classified as a case with lumbar disc herniation the worker should have been registered with the ICD-9 code 722.1 (1987–1996, “Displacement of thoracic or lumbar intervertebral disc without myelopathy”) or ICD-10 code M51.1 (1997–2010, “Lumbar and other intervertebral disc disorders with radiculopathy”). Patients can have several diagnosis and surgery codes at each admission. To be classified as a case in this study the primary diagnosis should be 722.1 or M51.1.

### Analyses

In total, there were 288,926 men included in the analysis (Table 1), exclusions are listed in “Appendix 1”.

**Table 1:** Age at the first health examination, smoking habits, weight and height in the construction workers according to the WBV exposure

	Construction workers		Foremen/white-collar workers
WBV exposure	No to low exposure	Moderate to high exposure	Reference
*n*	235,219	15,232	38,475
Age at health examination^a^	33.1 (12.8)	36.1 (10.8)	37.6 (11.4)
Weight^a^ (kg)	75.7 (10.1)	79.1 (11.1)	77.8 (10.0)
Height^a^ (cm)	177.5 (6.5)	177.2 (6.3)	178.6 (6.3)
Smoking habits^b^			
Non-smokers	97,074 (41.3)	4544 (29.8)	15,943 (41.4)
Ex-smokers	34,473 (14.7)	2646 (17.4)	6840 (17.8)
Smokers	89,215 (37.9)	6566 (43.1)	12,506 (32.5)
Unknown	14,457 (6.2)	1476 (9.7)	3186 (8.3)

^a^Mean, (SD)

^b^*n* (%)

The analysis was restricted to men as very few women had jobs that included high exposure to WBV. Persons who had emigrated or died before 1987 were excluded from the analysis. Weight and height from the first health examinations were used in the adjusted analysis and persons with missing information about weight or height were excluded. The analysis was restricted to men with a weight between 50 and 129 kg and a height between 150 cm and 199 cm and to persons with a BMI between 18.5 and 34.9 kg/m^2^. Smoking habits from the first visit was used if available and if missing data from the second or third visit was used.

The observation period was from January 1st 1987, until 31 December 2010 or death, emigration or the occurrence of hospitalization due to lumbar disc herniation, whichever came first. Person-years was calculated for each calendar year separately and stratified for age, weight, height and smoking habits. Poisson regressions were used to estimate relative risk with 95% CIs, adjusting for age (10-year classes 20–79 years of age), weight (20 kg classes, 50–129 kg), height (10 cm classes, 160–199 cm) and smoking habits (non-smokers, ex-smokers, smokers, unknown smoking habits). In the analyses, we used the categorized data which yields a dataset with count data for the different combinations of categories. Additional analyses restricted to ages with the highest incidence of lumbar disc herniation (30–49 years) were also performed.

## Results

In total, there were 2880 hospital admissions (~ 1% of study population) due to lumbar disc herniation among the male workers during the observation period (Table 2).

**Table 2:** Relative risk of hospitalization due to lumbar disc herniation according to exposure to WBV

Exposure groups	Cases (*n*)	Relative risk^a^ (95% CI)	
		Crude	Adjusted^b^
Referents^c^	293	1 (ref)	1 (ref)
None–very low (0–1)^d^	2417	1.30 (1.10–1.54)	1.23 (1.08–1.39)
Moderate–high (2–5)	170	1.41 (1.13–1.75)	1.35 (1.12–1.63)

^a^Estimated by poisson regression

^b^Analysis adjusted for age, height, weight and smoking habits; crude analysis only adjusted for age

^c^White-collar workers and foremen

^d^Low-exposure construction workers

Workers who were exposed to WBV at the time of the health examination had an increased risk of hospitalization compared to white-collar workers and foremen (Table 2). When this analysis was restricted to only include workers aged 30–49 years at the time of hospitalization the risk pattern became more evident (Table 3). When the analysis was further restricted to only include cases that occurred within 10 years from the health examination the risk pattern was similar (data not shown).

**Table 3:** Relative risk of hospitalization due to lumbar disc herniation at 30–49 years of age at first health examination according to exposure to WBV

Exposure groups	Cases (*n*)	Relative risk^a^ (95% CI)	
		Crude	Adjusted^b^
Referents^c^	121	1 (ref)	1 (ref)
None–very low (0–1)^d^	1539	1.48 (1.18–1.84)	1.47 (1.22–1.78)
Moderate–high (2–5)	121	1.69 (1.27–2.27)	1.69 (1.29–2.21)

^a^Estimated by poisson regression

^b^Adjusted for age, height, weight and smoking habits; crude analysis only adjusted for age

^c^White-collar workers and foremen

^d^Low-exposure construction workers

## Discussion

The findings in this study shows that workers exposed to WBV had an increased risk for hospitalization due to lumbar disc herniation.

There are a few previous studies indicating an association between WBV and back pain and lumbar disc herniation (Burström et al. 2015). Most previous research studied the 12-month prevalence of low back pain (Burström et al. 2015). Some MRI-studies have not been able to observe an association between exposure to WBV and degeneration or other pathology (Battie et al. 2002). For example, no differences in lumbar disc degeneration assessed with MRI (disc heights, bulges, herniations) were found between professional rally drivers, who are subjected to high WBV exposure, and a control population (Videman et al. 2000). Although disc degeneration is thought to be an important factor in the underlying pathology of disc herniation and LBP (Battie et al. 2009), disc degeneration and symptomatic disc herniation are different entities. Most studies on heritability and lumbar disc degeneration have been performed on asymptomatic individuals (Battie et al. 2009), and a weakness with this is that up to 70% of asymptomatic individuals have MRIs indicating a lumbar disc herniation (Boos et al. 1995). Thus, if the purpose is to study the consequences for the patient, clinical diagnoses considering pain seems more relevant than radiological diagnosis.

Several studies have used hospital discharge registers as an outcome measure of disease (Wahlström 2012; Heliövaara 1987; Kaila-Kangas et al. 2003; Mattila et al. 2008). Hospitalization indicates severe consequences of the disorder with considerable suffering for the patient, but only a minority of individuals with lumbar herniated disc or sciatica are hospitalized. A possible mechanism for the association between WBV and lumbar disc herniation is that WBV acts as a catalyst to further aggravate an already existing lumbar disc herniation. The relative risks would then be lower if cases of lumbar disc herniation with milder symptoms had been studied. Our study is the first study as far as we know that have used hospital discharge registers to study lumbar disc herniation and the association with exposure to WBV. Still, little is known about the long-term consequences for work–life participation, e.g., leaving the labor market in advance and this is an important area especially with the demographic situation in many developed countries with an ageing population and the increased awareness of the importance of maintaining people at work.

WBV may not just increase the incidence of lumbar disc herniation. A Dutch study observed that sickness absence due to intervertebral disc disorders lasted longer among workers exposed to WBV (crane operators) (Bongers et al. 1988). They also reported that workers with this diagnosis more often obtained a disability pension.

Experimental studies have investigated different aspects of WBV on the lumbar spine (Wilder and Pope 1996). The magnitude of vibration transmitted to the human spine is the greatest at resonant frequencies from 4.5 to 5.5 Hz and from 9.4 to 13.1 Hz. Bending and rotating postures increase vibration transmission, muscles are fatigued by vibration exposure, and oxygen consumption increases. Vibrations increase pressure within the lumbar discs and herniated discs have been produced in cadaveric lumbar motion segments exposed to vibrations (Wilder and Pope 1996). Wilder and Pope (1996) also suggests that prolonged vibration exposure may cause fatigue (cyclic damage) of spinal structures, similar to what occurs in mechanical structures through material fatigue. One hypothesis is that WBV, through one or several mechanisms described above, could contribute to increased degeneration of the discs and vertebrae in the back and thus increase the risk of subsequent symptomatic lumbar disc herniation. It has also been shown that age and sex have significant effects on fatigue strength in lumbar spinal bony injury (Schmidt et al. 2012). A majority of workers exposed to WBV are also exposed to prolonged sitting and different factors such as manual material handling and work with the back in stooped or twisted positions, factors that also increase the load in lumbar region and are associated with an increased risk of lumbar disc herniation (Seidler et al. 2003, 2009). The majority of studies on LBP and exposure to WBV have not been able to separate the contribution of these factors. Using data from JEM’s, we were able to adjust for back load and in an analysis on blue-collar workers, the results showed a 19% increased relative risk (95% CI 0.99–1.46) for moderate to high exposed workers compared to low exposed (Supplementary table S3).

Few studies have studied the relationship between lumbar disc herniation and exposure to WBV. Kelsey and colleagues published several papers in the 1970s, where increased risks were observed for disc herniation among professional drivers. None of the risk factors reached statistical significance, but the studies were rather small and this could explain the lack of statistically significant associations (Kelsey et al. 1984; Kelsey and Hardy 1975). More recent studies have observed associations between WBV and more severe LBP as assessed with pain intensity and the Roland and Morris disability scale score and one strength of those studies is the thorough exposure assessment (Bovenzi 2010; Bovenzi et al. 2006). In a meta-analysis by Bovenzi and Hulshof (1999), a summary prevalence odds ratio for sciatic pain of 2.0 (95% CI 1.3–2.9) for cross-sectional studies were reported for workers exposed to WBV compared to unexposed workers.

### Strengths and limitations

The prospective design of this study limits the risk for recall bias and the size of the study yields precise risk estimates. Another strength of the study was the outcome measure of hospitalization due to lumbar disc herniation based on the medical records after being admitted to a hospital ward. A selection bias could occur if physicians admitted subjects with high exposure to WBV more frequently than subjects with less-demanding jobs which we consider improbable. Hospital care is almost free of charge in Sweden and the referral is probably mainly depending on the severity of the pain and how well it can be moderated by pharmacological drugs. There could be some degree of misclassification in the register due to errors in coding or there could be wrong diagnosis by the physician. However, such misclassification would mainly be non-differential according to WBV and attenuate the association. Some of the workers in our reference group, for example, foremen, could previously have been working in a job that entailed exposure to WBV and this has not been taken into account in the analyses, but would lead to an underestimation of the risk. We were able to include back load, a measure of physical work demands such as heavy lifting and working in strenuous positions. When adjusting for back load in the analyses only including blue-collar workers an increased risk for high compared to low exposed workers was observed (Supplementary data, Tables S1–S4). This analysis showed a weaker association between WBV and hospitalization for lumbar disc herniation which is expected if exposure to WBV and back load is positively correlated. We have low power to estimate relative risks of WBV stratified for low back load and there is also the risk of over-adjusting statistical models. Thus, the findings should be cautiously interpreted.

We estimated the exposure from the first health examination and the exposure may have changed during time which may decrease the association and the analysis of ages 30–49 years (Table 3) showed slightly higher risk estimates when also including white-collar workers as a reference group. Exposure levels of WBV has decreased from the 1970s by improved dampening and driver’s seats. Nevertheless, it is still common with significant exposure to WBV with high acceleration levels.

The construction workers in this study had good access to occupational health, and medical care is almost free of charge in Sweden. Other factors associated with socioeconomic status are being overweight and smoking, but they were controlled for in the analyses. The major reason for hospitalization due to lumbar disc herniation is the pain or the occurrence of major neurological symptoms. Both the workers and the physicians may have different opinions of the need for hospitalization due to pain which should not lead to bias as long as it is not correlated to the exposure to WBV which we think is improbable.

Our results suggest that preventive measures to decrease exposure to WBV should continue. There is an European Directive on mechanical vibration with an action value of 0.5 m/s^2^ and a limit value of 1.15 m/s^2^ (EU 2002). Our results support measures to decrease high levels of WBV but our exposure estimates is too unprecise to estimate risks at the levels in the European Directive. Furthermore, exposure to high levels of WBV are mostly associated with prolonged sitting, strenuous working postures and heavy manual materials handling as in our study. Preventive measures to reduce the vibration acceleration levels and exposure durations will probably also reduce other factors related to lumbar disc herniation.

## Conclusion

This study further supports that occupational exposure to whole-body vibration increases the risk for hospitalization due to lumbar disc herniation.

### Electronic supplementary material

Below is the link to the electronic supplementary material.

Supplementary material 1 (DOCX 15 KB)

## Appendix 1

**Table:**

Exclusions	
All examined in the cohort	389,132
Women excluded (*N* = 19,418)	369,714
Excluding death or emigration before 1987 (*N* = 26,644)	343,070
Excluding persons shorter than 150 cm, taller than 199 cm and persons with a mass above 139 kg or below 50 kg (*N* = 3316)	338,485
Lack of information about WBV from JEM	49,559
Included	288,926
