# Supplementary material for: Exposure to whole-body vibration and hospitalization due to lumbar disc herniation
Source: Int Arch Occup Environ Health. 2018 May 31;91(6):689–94. doi: 10.1007/s00420-018-1316-5 (PMC6060752; doi:10.1007/s00420-018-1316-5)
Supplement: Supplementary file 1 — Supplementary material 1 (DOCX 15 KB) [file 420_2018_1316_MOESM1_ESM.docx]

**Supplementary results (Table S1 – S4)**

**Table S1.** Relative risk of hospitalization due to lumbar disc herniation according to exposure to WBV.

|  |  | Relative risk^a^ (95 % CI) | |
| --- | --- | --- | --- |
| Exposure groups | Cases (n) | Crude | Adjusted^b^ |
|  |  |  |  |
| Referents^c^ | 293 | 1 (ref) | 1 (ref) |
| None –very low (0-1)^d^ | 1635 | 1.26 (1.07-1.47) | 1.05 (0.57-1.90) |
| Moderate-high (2-5) | 129 | 1.33 (1.05-1.66) | 1.22 (0.65-2.30) |

1. Estimated by poisson regression.
2. Analysis adjusted for age, height, weight, smoking habits and back load; crude analysis only adjusted for age
3. White collar workers & foremen
4. Low exposure construction workers

**Table S2.** Relative risk of hospitalization due to lumbar disc herniation at 30-49 years of age at first health examination according to exposure to WBV.

|  |  | Relative risk^a^ (95% CI) | |
| --- | --- | --- | --- |
| Exposure groups | Cases (n) | Crude | Adjusted^b^ |
|  |  |  |  |
| Referents^c^ | 121 | 1 (ref) | 1 (ref) |
| None –very low (0-1)^d^ | 961 | 1.43 (1.14-1.78) | 1.38 (0.64-2.95) |
| Moderate-high (2-5) | 61 | 1.56 (1.12-2.17) | 1.69 (0.76-3.77) |

1. Estimated by poisson regression.
2. Analysis adjusted for age, height, weight, smoking habits and back load; crude analysis only adjusted for age
3. White collar workers & foremen
4. Low exposure construction workers

**Table S3.** Relative risk of hospitalization due to lumbar disc herniation according to exposure to WBV among blue-collar workers.

|  |  | Relative risk^a^ (95% CI) | |
| --- | --- | --- | --- |
| Exposure groups | Cases (n) | Crude | Adjusted^b^ |
|  |  |  |  |
| None –very low (0-1)^c^ | 1635 | 1 | 1 |
| Moderate-high (2-5) | 129 | 1.05 (0.86-1.28) | 1.19 (0.99-1.46) |

1. Estimated by poisson regression.
2. Analysis adjusted for age, height, weight, smoking habits and back load;
   crude analysis only adjusted for age
3. Low exposure construction workers

**Table S4.** Relative risk of hospitalization due to lumbar disc herniation at 30-49 years of age at first health examination according to exposure to WBV among blue-collar workers.

|  |  | Relative risk^a^ (95% CI) | |
| --- | --- | --- | --- |
| Exposure groups | Cases (n) | Crude | Adjusted^b^ |
|  |  |  |  |
| None –very low (0-1)^c^ | 961 | 1 | 1 |
| Moderate-high (2-5) | 61 | 1.10 (0.83-1.44) | 1.23 (0.94-1.62) |

1. Estimated by poisson regression.
2. Analysis adjusted for age, height, weight, smoking habits and back load;
   crude analysis only adjusted for age
3. Low exposure construction workers
